# Schwann Cell-Like Cells: Origin and Usability for Repair and Regeneration of the Peripheral and Central Nervous System

**DOI:** 10.3390/cells9091990

**Published:** 2020-08-29

**Authors:** Alois Hopf, Dirk J. Schaefer, Daniel F. Kalbermatten, Raphael Guzman, Srinivas Madduri

**Affiliations:** 1 Department of Biomedical Engineering, University of Basel, Gewerbestrasse 14, 4123 Allschwil, Switzerland; aloisc.hopf@unibas.ch (A.H.); Daniel.Kalbermatten@usb.ch (D.F.K.); 2 Department of Biomedicine, University Hospital Basel, Hebelstrasse 20, 4031 Basel, Switzerland; Dirk.Schaefer@usb.ch (D.J.S.); Raphael.Guzman@usb.ch (R.G.); 3 Department of Plastic, Reconstructive, Aesthetic and Hand Surgery, University Hospital Basel, University of Basel, Spitalstrasse 21, 4031 Basel, Switzerland; 4 Department of Neurosurgery, University Hospital Basel, Spitalstrasse 21, 4031 Basel, Switzerland

**Keywords:** Schwann cells, Schwann cell-like cells, human adipose stem cells, neurotrophic factors, peripheral nerve injuries, spinal injuries, brain injuries, axonal regeneration, myelin regeneration

## Abstract

Functional recovery after neurotmesis, a complete transection of the nerve fiber, is often poor and requires a surgical procedure. Especially for longer gaps (>3 mm), end-to-end suturing of the proximal to the distal part is not possible, thus requiring nerve graft implantation. Artificial nerve grafts, i.e., hollow fibers, hydrogels, chitosan, collagen conduits, and decellularized scaffolds hold promise provided that these structures are populated with Schwann cells (SC) that are widely accepted to promote peripheral and spinal cord regeneration. However, these cells must be collected from the healthy peripheral nerves, resulting in significant time delay for treatment and undesired morbidities for the donors. Therefore, there is a clear need to explore the viable source of cells with a regenerative potential similar to SC. For this, we analyzed the literature for the generation of Schwann cell-like cells (SCLC) from stem cells of different origins (i.e., mesenchymal stem cells, pluripotent stem cells, and genetically programmed somatic cells) and compared their biological performance to promote axonal regeneration. Thus, the present review accounts for current developments in the field of SCLC differentiation, their applications in peripheral and central nervous system injury, and provides insights for future strategies.

## 1. Introduction

**Figure 1:**
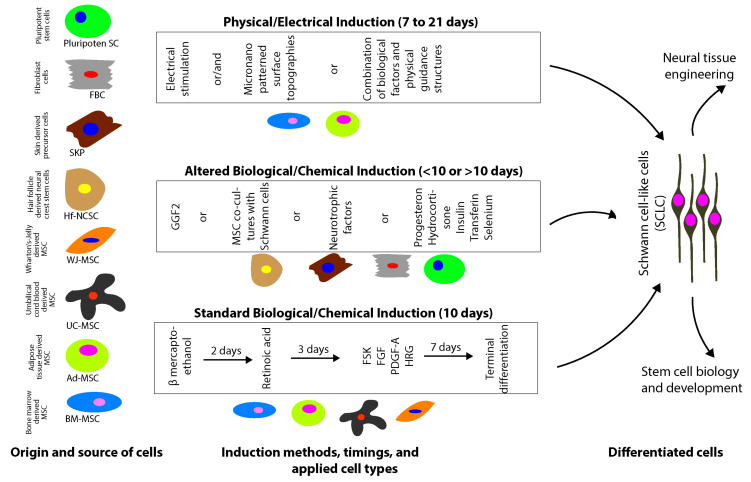
Mesenchymal stem cell (MSC) of different origin, current developments in the differentiation of MSC into Schwann cell-like cells (SCLC) and potential applications of SCLC.

Every year about 1 million people suffer from peripheral nerve injuries (PNI) worldwide [1,2]. In the case of simple nerve transection, end-to-end suturing is sufficient. However, long-gap nerve injuries that are not amenable with end-to-end suturing result in a significant clinical challenge. For this, autologous nerve transplantation is the current clinical gold standard [1,2], where the regenerating axons are supported optimally by endogenous physical and biological guiding scaffold. However, autologous nerve grafts are associated with several drawbacks, such as limited donor sites, modality mismatch, and co-morbidities, i.e., neuroma formation [3,4,5]. Within this context, bio-engineered nerve grafts combining physical guidance structures with neurotrophic cells, guidance cues, and signaling molecules provide an innovative and viable option for treating PNI [6]. There is growing evidence for the therapeutic potential of Schwann cells (SC) transplantation for promoting axonal regeneration and myelination in the peripheral and central nervous system (CNS) following injury [7,8]. In spite of the promising outcome, the harvest of autologous SC represents almost the same limitations that are associated with autologous nerve grafting, i.e., healthy nerve surgical harvest and related functional impairment [9]. Further isolation, culture, and purification has been shown to be challenging due to the limited expansion potential of SCs and frequent contamination with rapidly proliferating fibroblasts [10,11,12,13]. Therefore, a viable option would be to generate Schwann cell-like cells (SCLCs) from different sources with reduced limitations [10]. Thus, the need for stem cell-derived SCLC has evolved. For this, cells with self-renewal capacity, multi-lineage potential, and low immunogenicity are highly suitable. Additionally, cells that are easily accessible with abundant quantities become furthermore attractive. Thus, there is a great need for developing new strategies for the generation of therapeutic SCLC using stem cells of different origins (Figure 1 and Table 1).

### 1.1. Schwann Cell Development and Homeostasis

SCs are the glial cells of the peripheral nervous system (PNS), named after Theodor Schwann, one of the founders of the cell theory. Ramon y Cajal in 1928 concluded, among others like Ranvier and Waller, that axonal recovery in the PNS is a result of axo-glial bidirectional interaction [14]. Nowadays, SCs are recognized as one of the largest, ultra-structurally most complex cells in the body. However, they are still capable of rapid transformation in development and injury [15]. SCs originate from migrating neural crest cells. In vivo differentiation of neural crest into the SC lineage has not been fully elucidated. However, it is known that the transcription factor SRY-Box Transcription Factor 10 (SOX10) is an essential master regulator for generating the earliest cells in the SC lineage, as reviewed by Mirsky in 2008 [16]. During the development, SCs are associated with a bundle of axons and release a variety of neurotrophic factors, such as nerve growth factor (NGF), brain-derived neurotrophic factor (BDNF), glial cell line-derived neurotrophic factor (GDNF), and neurotrophin 3 (NT3), which are involved in axonal growth and pathfinding. By proliferation and extensive extension of SC structures, axons are segregated into smaller bundles in a process called radial sorting. Most of the small-diameter axons, including many sensory and autonomous axons, remain in such bundle associated with non-myelinating Remak SCs. In contrast to the CNS, single Remak SC wraps a single axon in PNS. Remak SCs remain proliferative throughout life and express several markers typically found in developing SCs, such as neural cell adhesion molecule (NCAM), p75 neurotrophin receptor (p75NTR), and glial fibrillary acid protein (GFAP) [17,18]. Radial sorting of large-diameter axons, including some sensory and many motor axons, proceeds until one SC surrounds one axon. Such SCs wrap myelin structures around the axons, resulting in the formation of mature myelin sheath [19]. Differentiation of myelinating SCs is controlled by the Krox20 transcription factor (Egr2), as evidenced by the inability of Krox20 deficient mice to form myelin sheaths in vitro and In vivo [15,20,21].

### 1.2. PNS Injury

SCs distal to the injury site lose their contact with axons following injury and undergo significant changes in their signaling environment due to missing contact with axonal-derived factors. Macrophages invade the injury site in large quantities and release a wide range of cytokines that will further influence the SC [10]. The hypoxic environment within the damaged nerve induces vascular endothelial growth factor A (VEGF-A) secretion by macrophages, resulting in the polarized vascularization, which, in turn, guides the SCs to bridge the nerve gap [22]. Following these changes within the injury microenvironment, the fully differentiated non-myelinating Remak SC as well as the myelinating SC converse to a repair SC phenotype [23]. Thus, the SC phenotype transition activates cellular mechanisms resembling developmental molecular sequences, such as up-regulation of neurotrophic factors (NTF), i.e., NGF, BDNF, ciliary neurotrophic factor (CNTF), NT3, extracellular matrix (ECM) proteins (laminin 1 and 2, and fibronectin), and NCAM, for regulating neuronal survival and axonal regeneration [24,25,26,27]. Further, SCs regulate self-renewal and survival by autocrine signaling, e.g., insulin-like growth factor 2 (IGF-2), platelet-derived growth factor (PDGF-BB), neurotrophin-2 (NT2), and leukemia inhibitory factor (LIF) [28]. By cellular elongation and branching, SCs form so-called bands of Büngner, “cellular tracks” in which axons can regenerate. This transition is further required for myelin autophagy and to secrete cytokines that attract the macrophages for later stages of myelin clearance [29]. These repaired SCs navigate the regenerating axons and remain functioning for a long time, often for months or even years in humans due to the slow axonal growth (i.e., <3 mm/day) [21]. Further, several studies have demonstrated that SC transplantation support functional axonal outgrowth in vitro and In vivo following injury [30,31,32].

### 1.3. CNS Injury

Myelin debris, astrocytes, and oligodendrocytes (OC) collectively become strong inhibitors of axonal regeneration in the CNS following injuries [33]. Myelin in the CNS is produced by OCs in contrast to PNS and contains inhibitory molecules, such as Nogo, OC-myelin glycoprotein, and myelin-associated glycoprotein (MAG). These molecules bind to Nogo receptors on the distal tip of regenerating axons to transmit inhibitory signals [34]. In contrast to SCs in the PNS, OCs in the CNS depend on an axonal-derived signal for survival. Therefore, OCs undergo apoptosis or enter a quiescent state after injury [35], resulting in reduced myelin clearance in the CNS. Remaining myelin can be found up to 22 months post-injury in a rat optic nerve injury model [36]. In addition to the uncleared myelin and associated inhibitory molecules, astrocytes start proliferating and extend their processes, resulting in the formation of astroglial scar that inhibits axonal regeneration physically and chemically [37,38]. One of those, monocyte chemotactic protein-1 (MCP-1), promotes the recruitment of proinflammatory macrophages, releasing tumor necrosis factor α (TNF-α) and inducible nitric acid synthase (iNOS) [39]. TNF-α increases local expression of caspases, leading to apoptosis, and iNOS promotes the apoptosis of the damaged neuron. This process becomes inhibitory for the axonal regrowth over a prolonged period [40,41]. However, spontaneous regeneration of myelin sheaths often occurs following CNS demyelination, mainly by the differentiation of oligodendrocyte precursor cells (OPC) into myelinating OCs or SCs. Normally SCs are neither present in CNS nor migrate into the CNS due to mutual exclusivity of SCs and astrocytes [42,43]. Based on the earlier findings revealing the SCs presence and their myelin regeneration within the niche of spinal cord injury (SCI) of rodents as well as humans, it was concluded that SCs migrate into SCI niche from the periphery after the disruption of the astrocyte-SC exclusivity [42,44,45,46,47]. However, recent findings suggest that SCs from the PNS rarely enter the remyelinating spinal cord, but the vast majority of the SCs are derived from endogenous OPC [48,49]. The signals instructing the OPC differentiation into myelinating OC or myelinating SC are released by reactive astrocytes. Within this context, the inhibition of astrocyte activation has resulted in enhanced SC-myelination in contrast to OC-myelination [50]. Following demyelination, activated OPC and endothelial cells release ligands for bone morphogenetic protein (BMP) and Wnt signaling pathways, whereas reactive astrocytes release the BMP/Wnt antagonist Socstdc1. The BMP/Wnt signaling balance instructs OPC fate decisions shortly after activation. In the absence of Socstc1, OPCs differentiation into SCs is favored within the astrocyte-free zone [51].

Chronic stage SCI in humans often results in schwannosis, which is an aberrant growth of SCs and nerve fibers in the CNS. Schwannosis impedes effective axonal outgrowth and promotes aberrant axonal growth, leading to pain, spasticity, and other abnormal responses in the patients suffering from chronic SCI [52]. In more than 80% of cases, patients with chronic SCI (i.e., >4 months) exhibit schwannosis in contrast to acute SCI [53]. Therefore, gaining a better understanding of endogenous SCs regulation may help developing new treatment strategies.

### 1.4. SC Transplantation

#### 1.4.1. SC Transplantation in the PNS

Seddon first described autologous peripheral nerve grafting in 1947. He was the first to use autologous sensory nerve grafts for bridging the gaps in the peripheral nervous system [1], although this procedure is associated with important drawbacks, such as sensory loss at the donor site, neuroma formation, and lack of sufficient graft material. Nonetheless, autologous nerve grafting has become the gold standard to treat long gap peripheral nerve injuries. There is a growing interest in the field for developing a viable alternative, as evidenced by 11 Food and Drug Administration (FDA)-approved nerve conduit devices and several in further development [2]. Although nerve conduits appear to enhance nerve regeneration in short gaps, it is increasingly difficult for supporting long-gap nerve injuries [3]. Therefore, functionalizing the nerve conduits with a variety of growth-promoting substrates has been widely considered for enhancing their biological function [4]. Within this context, the first successful SC transplantation in a rat model was demonstrated back in 1992 by Guenard et al. Transplantation of in vitro expanded SCs results in the axonal regeneration and myelin formation in a sciatic nerve injury model [5]. Further, the nerve conduit seeded with human SCs supports repair and regeneration of a 5 mm nerve gap injury in mice. Interestingly, transplanted human SCs survive for at least 6 weeks and form myelin around the regenerating axons [6]. Since then, several studies have demonstrated the benefits of SC transplantation for peripheral nerve regeneration in the variety of animal models. Applications of SCs and nerve conduits for treating nerve injuries was recently reviewed by Han et al., in 2019 [7]. Furthermore, successful autologous SC transplantation in humans was reported for the first time in 2016. Human SCs are isolated from the sural nerve as well as from the injured sciatic nerve stump and combined with a sural nerve graft to repair a 7.5 cm long sciatic nerve. For this, the cells are expanded in vitro for 7 days and incorporated into an autologous sural nerve graft for implantation. Patients treated with SCs-enriched nerve grafts restore significant sensory and motor functions, indicating the safety and feasibility of SCs clinical translation [4].

#### 1.4.2. SC Transplantation in the CNS

An attractive option for CNS regeneration is to adopt and acquire the favorable properties of peripheral nerves, i.e., grafting of SC to revert scar formation and to promote axonal regeneration. Back in 1928, Ramon y Cajal, for the first time, demonstrated the potential of peripheral nerve transplantation to regenerate spinal axons. Further, he suggested that SCs could be used to overcome the non-permissive environment and to enable axonal regeneration [14]. In 1975, when techniques for glial cell isolation and purification were developed, Richard Bunge suggested the use of purified SC for the repair of the central nervous system. Richardson et al. in 1980 demonstrated the spinal axonal ingrowth into the transplanted peripheral nerve graft using an adult rat SCI model [54]. Since then, the beneficial effects of SC transplantation have been demonstrated by several studies for the repair and regeneration of axons in the CNS [55,56,57]. Transplanted SCs are capable of promoting remyelination and functional restoration [45,58]. As described previously, SCs express a multitude of factors, such as NGF, NT3, BDNF, fibroblast growth factor (FGF), GDNF, and CNTF, as well as ECM proteins—laminin and fibronectin, which are crucial for axonal growth and elongation [59,60]. In contrast to OCs in the CNS, SCs produce myelin sheaths, which is unlikely to be the target of an autoimmune reaction underlying autoimmune disease, such as multiple sclerosis (MS). In 2001, a phase 1 clinical trial was initiated at Yale University for treating five MS patients with SCs. However, the trial was terminated after the third patient due to the lack of evidence for the therapeutic benefits (myelination) of transplanted SCs [61]. Moreover, the inability of post-natal SC to migrate through normal white matter or astrocytes rich areas, such as glial scars, further limits the SCs therapy for MS patients [62]. Within this context, embryonic SC precursor cells exhibit enhanced migration to demyelinated lesion sites in a rat model and improve myelin regeneration [63]. In an alternative approach, genetic modification of adult SCs’ adhesion properties results in effective migration, which, in turn, improves myelin regeneration and functional restoration in rats with SCI [64,65]. Thereby, modified SCs hold the potential for demyelinating diseases [42]. Even though there is growing evidence for the therapeutic potential of SCs for treating CNS, their clinical transition is still challenging. As described by Bunge in 2016, the need for combinatorial strategies has emerged for treating SCI due to the secondary tissue damage, cell death, inflammation, scar formation, inhibitory factors, and silenced axons [66]. Within this context, various co-treatment strategies were developed in the Bunge lab in an attempt to increase the therapeutic efficacy of SC for CNS repair. Those include steroids, neurotrophins, enzymes, cyclic AMP, and olfactory ensheathing cells [67,68,69,70,71]. Furthermore, genetically modified SCs for their neurotrophic potency have resulted in increased neurotrophin release and enhanced functional axonal regeneration [72,73,74]. Thus, the combinatorial treatment approaches have positively increased the quality and quantity of axonal regeneration, remyelination, and functional recovery, i.e., loco-motor outcome of paralyzed rats [66]. These topics were reviewed in great detail by Fortun et al., in 2009, Tetzlaff et al., in 2011, and Griffin et al., in 2020 [75,76,77].

### 1.5. Biomaterial/Scaffolds

Effective regeneration of severed nerves largely depends on the injury size. The end-to end suturing is feasible for small gap injuries. However, there exists a critical nerve gap length, allowing neither spontaneous regeneration nor end-to-end suturing. End-to-end suturing of large nerve gaps (>3 mm) leads to tension between the nerve segments and is often associated with poor outcomes [78]. Implantation of nerve auto or allograft can bridge such large gap injuries. However, these are coming with the aforementioned drawbacks, such as donor site morbidity, functional impairment, and immunological complications in the latter case. Therefore, there is an increased research focus on developing artificial nerve conduits. An ideal nerve conduit incorporates several attributes, such as biocompatibility, biodegradable, flexibility, stability, and bio-inspired functional design. Sarker et al. recently reviewed advancements in the field of artificial nerve conduits. In this review, varying structures from simple hollow tubes to complex conduits incorporating cells and bioactive molecules mimicking the autologous nerve were detailed and discussed. Shortly, it concludes that cell loaded nerve conduits possess superior biological performance over hollow structures, and SCs outperform among a wide variety of cells that are under investigation for nerve regeneration [79].

### 1.6. Immunosuppression Following PNI and SCI

Considerable evidence has been shown for the therapeutic potential of SC and stem cell transplantation in PNI and SCI. However, the downside of using non-autologous cells is immune rejection. Therefore, the efficiency of non-autologous cell transplantation relies on effective immune-suppressive drugs. Several studies have evaluated tacrolimus (FK506) and cyclosporine (cyclosporine A or CsA) as main adjuvants for stem cell transplantation. Tacrolimus is commercially available and is widely used for transplantation procedures. Its mode of action relies primarily on inhibition of T-lymphocyte proliferation. Similar to tacrolimus, cyclosporine inhibits T-cell activation. It has been shown that systematic cyclosporine, as well as tacrolimus administration, significantly reduces T-cell infiltration into allografts, resulting in the enhanced therapeutic efficacy of transplanted cells in PNI and SCI [80]. Furthermore, it has been shown that tacrolimus induces SC proliferation, which, in return, triggers axonal regeneration [81]. Sosa et al. reviewed the neuroprotective effects and functional recovery after PNI and SCI in response to cyclosporine and tacrolimus, in 2005 [82]. Numerous preclinical studies have investigated the use of tacrolimus and cyclosporine in an allogeneic stem cell transplantation for treating SCI, as reviewed by Antonios et al. in 2019 [41].

## 2. Origin and Therapeutic Effects of Schwann Cell-Like Cells (SCLC)

### 2.1. Mesenchymal Stem/Stromal Cells

Human mesenchymal stem/stromal cells (MSCs) are defined by the International Society for Cellular Therapy by three minimal criteria: (1) adherence to plastic; (2) >95% of cells must be CD105+, CD73+, or CD90+, as well as <2% CD45+, CD34+, CD14+, CD19+, major histocompatibility complex II (MHC II) positive; (3) multipotent differentiation potential [83]. MSCs with self-renewal capacity, high proliferation ability, multilineage potential, and neurotrophic potency hold promise for the clinical treatment of nerve injuries. For the first time, MSCs were discovered in the bone marrow (BM), which is still the most studied MSC source. Later on, MSCs were isolated from the variety of tissues, including adipose tissue, umbilical cord tissue, Wharton’s-jelly, hair follicles, and skin. Preferably, these cells should be easily accessible in abundant quantities for clinical applications. Nowadays, next to BM, the most preferred MSC source is adipose tissue due to their abundance per gram tissue and easy accessibility [84]. However, umbilical cord tissues, such as cord blood and Wharton’s Jelly, enable non-invasive isolation procedures, thus making them an attractive source for MSCs (Figure 1). A wide range of MSCs is currently under clinical trials with a major focus on implantation techniques, safety, and efficacy. Even though preclinical studies encourage the use of MSCs for treating human SCI, the outcome of clinical trials remain controversial, as described by Soria-Zavala et al. in 2020 [85].

#### 2.1.1. Biological/Chemical Induction

Dezawa et al. described the differentiation of rat MSCs into SCLC in vitro for the first time in 2001 [86]. This original protocol by Dezawa was developed for the differentiation of rat bone marrow-derived MSC (BM-MSC) into SCLCs. Shortly, sub-confluent MSCs are incubated in alpha-MEM containing 1 mm beta-mercaptoethanol (BME) for 24 h. BME is a reducing agent and is known to induce neurite-like processes in MSC culture, previously used to induce neuronal differentiation [87]. Then, the media is removed, and the cells are washed with phosphate-buffered-saline (PBS), followed by 72 h of incubation in alpha-MEM media supplemented with 10% FBS and 35 ng/mL all-trans-retinoic acid (RA). RA regulates the expression of various transcription factors during early neuronal differentiation and increases the responsiveness to neurotrophins [88]. After a PBS wash, MSCs are transferred to alpha-MEM supplemented with 10% FBS, 5 µM forskolin (FSK), 10° ng/mL recombinant human basic-fibroblast growth factor (bFGF), 5 ng/mL recombinant human platelet-derived growth factor-AA (PDGF-AA), and 200 ng/mL recombinant human heregulin-beta1 (HRG) for 7 days. PDGF-AA, bFGF, and HRG are neurotrophins involved in the differentiation and proliferation of glial cells (SC). Further, bFGF and PDGF-AA are potent mitogens for MSCs. In the meantime, HRG is found to induce neural crest cells selectively into SC [89,90]. FSK increases the level of intracellular cyclic adenosine monophosphate (cAMP) [91]. Further, the cAMP elevation is known to increase responsiveness to neurotrophic factors. Thus, the induction process is a result of the potential synergistic effect of bFGF, PDGF-AA, HRG, and FSK [86,92]. Based on the protocol by Dezawa, several derivatives are developed over time using MSCs of different origins. Improved differentiation of MSCs is achieved by adding the glial growth factor (GGF-2), which is a potent SC mitogen that stimulates peripheral nerve regeneration and restricts neural crest stem cell (NCSC) differentiation to the glial lineage [93,94,95,96]. Further, co-cultures of BM-MSC and adipose-derived MSC (Ad-MSC) in the presence of primary SCs result in the differentiation and expression of the SC markers—peripheral myelin protein 22 (PMP-22) and S100—for up to 12 days (Figure 1 and Table 1) [97].

#### 2.1.2. Physical-Electrical Induction

Another approach, which was mainly studied for the differentiation of neural stem cells (NSC) into neurons, is electrical stimulation for the differentiation of MSCs into SCLCs [98,99,100]. Electrical stimulation only is successful in inducing MSCs into SCLCs using a flexible, highly conductive (sheet resistance < 1 kΩ/sq) inkjet-printed graphene interdigitated electrode circuit. Following electrical stimulation, the expression of the growth factors, i.e., NGF, GDNF, and BDNF, is up-regulated in comparison to chemical induction method. Further, electrically-induced MSCs show a high level of phenotypic markers specific for SC, i.e., p75, S100, and S100β, compared to chemically-induced and naïve MSCs. The possible mechanism of differentiation by electrical stimulation is assumed to be associated with altering the cellular membrane potential through hyperpolarization and depolarization, modifying ion channel density, receptor distribution, and calcium channel activation [101]. Further, it has been shown that various signaling pathways, i.e., mitogen-activated protein kinase (MAPK), Phosphoinositide 3-kinases (PI3K), and Rho-associated protein kinase (ROCK) pathways, regulating MSC’s proliferation and differentiation, are activated in response to electrical fields [102,103,104]. Another approach involving biomechanical forces or micro-nano patterned topographical surfaces has demonstrated the feasibility of controlling the fate of the stem cells. MSC cultured on imprinted SC topographies results in the direct differentiation into SCLC [105]. On the other hand, combining a micro-patterned substrate with chemical induction does not improve the differentiation process. Although the micro-pattern has a significant effect on cell alignment and elongation of the differentiated cells, the percentage of SCLC is not affected [106]. However, it is predicted that biophysical forces and mechanotransduction play a fundamental role in instructing the cell fate. It has been demonstrated that physical cues play an important role in embryonic stem cell (ESC) differentiation in vitro [103]. For example, shear stress is linked with ESC differentiation towards vascular endothelial cells, and the stretching of MSCs results in the up-regulation of smooth muscle cell markers [107,108]. Thus, the physical cues and structural features have gained increasing focus in the field, highlighting their important role in cell differentiation and transplantation (Figure 1 and Table 1) [105,109].

#### 2.1.3. Bone Marrow-Derived MSC

##### In Vitro Characterization

MSCs were isolated for the first time from BM, and these cells were extensively studied for various applications, as recently reviewed by Gomez-Salazar et al. in 2020 [110]. As shown for rat BM-MSCs, human BM-MSCs have the potential to differentiate into the glial lineage and express typical glial markers like S100B, GFAP, p75, and erbB3. Early morphological changes are observable within 4 to 5 days in the presence of differentiation media supplemented with GGF-2. BM-MSC morphology changes from flat, fibroblastic phenotype to a bipolar, elongated spindle-shaped, which is an SC’s characteristic phenotype [111]. Human BM-MSCs-derived SCLCs promote neurite sprouting from rat dorsal root ganglion (DRG) neurons in vitro [111]. Further, BM-MSCs-derived SCLCs render the microenvironment more favorable for tissue repair by releasing various growth factors, such as VEGF-A and hepatocyte growth factor (HGF) (Figure 1 and Table 1) [112].

##### Application in the PNS

A crucial function of SCs in PNI regeneration is their ability to remyelinate the regenerating axons. Even if the axons would reach their target, proper myelination is crucial for normal neuronal function and conduction speed [109,113]. Therefore, Dezawa et al. transplanted BM-MSCs-derived SCLCs with a GFP marker within a 15 mm hollow conduit (Amicon, Beverly, MA, USA) using a nerve gap-injury model. Within 3 weeks, successful nerve regeneration, along with newly formed myelin structures, is visualized by GFP expressing cells [83]. Significant improvement in functional and behavioral recovery (gait analysis) is observed after 6 months [114,115]. In another study, transplantation of 10 mm hollow conduits (Amicon, Beverly, MA, USA) seeded with human BM-MSCs-derived SCLCs and tacrolimus co-treatment results in the recovery of sciatic nerve function, as measured by walking track analysis in rats within 3 weeks [116].

##### Application in the CNS

Rat BM-MSCs-derived SCLCs promote locomotor and sensory function when grafted into SCI through 3 mm atelocollagen honeycomb (Koken Inc., Tokyo, Japan) scaffold in comparison to cell-free scaffolds [117]. Follow up studies have further revealed the improvement in anatomical and functional features of regenerated spinal cord tissue in response to BM-MSCs and BM-MSCs-derived SCLCs. However, undifferentiated BM-MSCs better support axonal regeneration, while BM-MSCs-derived SCLCs promote significant remyelination. Therefore, the authors suggest that a combination of SCLCs and BM-MSCs may become effective in treating SCI [118]. The application of human-MSC-derived SCLCs in SCI is still lacking. However, it has been shown that human-MSC-derived SCLC supports axonal outgrowth in an ex-vivo SCI model by secreting HGF and VEGF [112].

##### Limitations

To overcome the shortfall in terms of phenotypical stability of SCLCs, neuroectodermal progenitors from human BM-MSCs are selectively expanded and induced into SCLCs via an intermediate neurosphere. For this, a sphere-forming protocol used for skin precursor cells (SKPs) is adapted by Dezawa et al. in 2001 to foster the expansion of neuroglial progenitors within the BM-MSCs population [86,119,120]. Resulting SCLCs promote axonal outgrowth and myelination in vitro. Implantation into a rat PNI model involving 16 mm long chitosan conduit reveals the formation of human myelin basic protein (MBP) and compact myelin sheath after 8-weeks. The rats are co-treated with cyclosporine A [121]. However, BM-MSCs possess important limitations for their clinical transition. Firstly, the isolation of BM-MSCs is an invasive and painful procedure. Secondly, the ratio of MSCs in the bone marrow is relatively low (<1/100,000), and lastly, the quantity of bone marrow that can be harvested from patients is strictly limited [122]. Thus, there is a need for an alternative viable source of cells.

#### 2.1.4. Adipose Tissue-Derived MSC

##### In Vitro Characterization

Compared to BM-MSCs, Ad-MSCs are easily accessible from patients in abundant quantities (i.e., 500 times higher cell count). Further, it has been shown that Ad-MSCs possess rapid proliferation capacity and immune-privileged [123,124,125]. Kingham et al., using rat cells, reported the first successful differentiation of Ad-MSCs into SCLCs. For this, they used the previously established Dezawa’s protocol with a slight modification of increasing induction time and concentration of FSK and GGF-2 [126]. Rat Ad-MSCs-derived SCLCs are well studied for their expression of a neuroglial marker, neurotrophic factors, neurotransmitter, and related receptors. Their potential to promote axonal regeneration and myelin formation has been demonstrated by several studies in vitro and in vivo [127,128,129,130,131,132]. Differentiation of human Ad-MSCs into SCLCs results in the change of morphology from flat, fibroblast-like structure to elongated, spindle-shape, resembling the primary human SC. The secretion of GDNF, NGF, BDNF, VEGF-A, and angiopoietin-1 proteins is found to increase in the differentiated Ad-MSCs in vitro [133,134]. Ad-MSCs-derived SCLCs enhance neurite outgrowth from DRG neurons in vitro (Figure 1 and Table 1) [133].

##### Application in the PNS

Self-aligned rat Ad-MSCs-derived SCLCs in a collagen matrix support the axonal regeneration in a 15 mm rat PNI model. After 8 weeks, a 3.5-fold greater amount of axons is observed in conduits with SCLC than in cell-free conduits [135]. Further, rats treated with SCLC-seeded fibrin or silicon conduits following gap-injury exhibit improved nerve regeneration and functional outcome postoperatively at 2 weeks [128], 16 weeks [136], and 6 months [137]. Transplantation of human SCLCs into a rat tibial nerve crush injury supports the axonal regeneration and enriches the distal nerve with regenerating axons and MBP-positive myelin structures after 8 weeks of implantation [134]. Tubular fibrin conduit loaded with human Ad-MSCs-derived SCLCs results in enhanced angiogenesis and early nerve regeneration within 2 weeks in a rat 10 mm sciatic nerve injury model that is co-treated with cyclosporine A [133].

##### Application in the CNS

Collagen scaffolds loaded with Ad-MSC-derived SCLCs significantly enhance locomotor and sensory scores in rats with 3 mm hemisection SCI in comparison to cell-free implants. Further, a comparison of functional outcomes between BM-MSC-derived SCLCs and Ad-MSC-derived SCLCs reveals no significant difference, suggesting their comparable therapeutic performance [138]. In a 3 mm deep brain contusion, it has been shown that Ad-MSC-derived SCLCs improve behavioral performance after 30 days of implantation [139]. These results prove the ability of Ad-MSC-derived SCLCs to survive and exert their therapeutic function, i.e., neuronal survival, axonal regeneration, and remyelination within the microenvironment of CNS injury.

##### Limitations

Withdrawal of differentiation media from human SCLCs results in the rapid reversal of the SCLCs phenotype to stem cell-like characteristics [140]. These observations suggest that the differentiation process is reversible, and the long-term stability of SCLC is subjective to the constant availability of differentiation factors. GGF-2 is a key axonal-derived factor for SC maintenance In vivo, while SCs release BDNF and GDNF for neuronal maintenance [26,141]. Stimulation of Ad-MSCs with differentiation media containing a high concentration of GGF-2 mimics paracrine signaling and eventually results in BDNF and GDNF expression. However, the increased expression of GDNF and BDNF and the reduced NT-3 expression in response to GGF-2 stimulation may not indicate true differentiation. In an attempt to improve the quality of the differentiation process for human Ad-MSCs (stability and functional characteristics), the protocol by Dezawa et al. is modified and additional factors, i.e., progesterone (Prog), hydrocortisone, and insulin-transferrin-selenium, are added [142,143]. SCLCs, resulting from the modified protocol, exhibit enhanced performance in vitro and In vivo. Collagen sponge loaded with human SCLCs is implanted into a 10 mm sciatic nerve gap, and the outcome analysis reveals the enhanced stability (long-term survival), proliferation, myelination, and improved motor function within 4 months. Experimental rats are immunosuppressed by cyclosporine A [143]. However, the heterogeneity of stromal-vascular fraction (SVF)-derived Ad-MSCs represents the important limitation for their therapeutic efficiency. For effective clinical applications and reproducibility, it would be crucial to identify specific subpopulations within the SVF Ad-MSC pool. Furthermore, it is worth comparing the therapeutic performance of the cells resulting from the following two different strategies; 1) High purity Ad-MSC-derived SCLCs and 2) In vivo transdifferentiation of Ad-MSC into SCLCs in response to the localized release of growth factors.

#### 2.1.5. Umbilical Cord-Derived MSC

MSCs can be isolated from various tissues that are generated during childbirth, i.e., umbilical cord blood, placenta, perivascular tissue, amniotic fluid, and Wharton’s jelly (tissue surrounding the umbilical cord vessels). The isolation from these tissues is easier, non-invasive, and economical than bone marrow aspirate or adipose tissue [144]. Therefore, these tissues represent a potential alternative to Ad-MSCs and BM-MSCs involving invasive procedures. MSCs from the umbilical cord can be harvested without risk for either mother or child and cryopreserved [145]. Interestingly, Umbilical cord derived MSCs (UC-MSCs) are currently subject of dozens of clinical trials for various diseases, including spinal cord injuries. However, in-vitro UC-MSCs-derived SCLCs are not yet a subject of a clinical trial. But the usage of naive UC-MSCs shows safety, survival, and integration capabilities in human patients, as reviewed by Couto et al. in 2019 (Figure 1 and Table 1) [146].

#### Umbilical Cord Blood-Derived MSC

##### In Vitro Characterization

Human umbilical cord blood-derived MSCs (UCB-MSCs) differentiation into SCLCs consists of a two-step process. First, UCB-MSCs are induced into free-floating neurospheres that are positive for nestin while being negative for GFAP and S100. Further differentiation of neurospheres into SCLCs is achieved by treating with RA, FSK, bFGF, PDGF-AA, and HRG, as described by Dezewa et al. Resulting cells do express the SC markers—S100 and GFAP—and support neuronal differentiation and axonal outgrowth in vitro. However, UCB-MSCs-derived SCLCs begin to revert to a flat morphology after passage three, indicating the need for the continuous support of the differentiation factors [147]. On the other hand, direct differentiation of UCB-MSCs into SCLCs is achieved by the previously established protocol by Dezawa et al. For this, pretreatment with BME and bFGF is followed by RA treatment and differentiation process involving FSK, bFGF, PDGF-BB, NGF, and HRG. Notably, the composition of the differentiation media is adapted to the new cell origin by including PDGF-BB in the place of PDGF-AA [148]. In contrast to PDGF-AA, PDGF-BB plays an important role in actin reorganization [149]. Furthermore, NGF is also added to the media, which is shown to promote neural precursor cell differentiation into mature neurons and glial cells in vitro [148,150].

##### In Vivo Application

The biological performance of UCB-MSCs-derived SCLCs still remains to be elusive for treating PNI or SCI. However, undifferentiated UCB-MSC transplantation into a sciatic nerve crush model results in enhanced BDNF and TrkB expression and improved functional recovery [151]. Transplantation following a spinal cord contusion injury by weight drop reveals the survival and differentiation of human UCB-MSCs into neurons, OCs, and astrocytes, resulting in the enhanced functional recovery [152,153]. Therefore, we hypothesize that in vitro differentiated UCB-MSCs may have a beneficial effect on neuronal survival, axonal regrowth, remyelination, and functional restoration in PNS and CNS.

##### Limitations

The vast abundance, availability of donors, and reliability of sample collection make UCB-MSCs be highly promising cell source. However, the clinical applicability of UCB-MSC-derived SCLCs is limited due to the long two-step differentiation process and lack of studies demonstrating their therapeutic capacity In vivo. In addition, functional and phenotype stability of transplanted cells are considered to be crucial for maintaining the safety and efficacy. Moreover, UCB-MSCs are isolated from the umbilical cord; therefore, their application as an allograft for many sections of the patients is obvious, and thus, it is inevitable to follow immune suppression procedure. However, UCB-MSCs are less mature than other types of adult stem cells, indicating their low-immunogenicity [154].

#### Wharton’s-Jelly-Derived MSC

##### In Vitro Characterization

Human Wharton’s-Jelly-derived MSCs (WJ-MSCs) can be differentiated into SCLCs using the protocol established by Dezawa et al., with minor modifications. Within eight days, WJ-MSCs change to an SC-like morphology. From large and flat WJ-MSC morphology, they change to a bi-polar spindle-shaped morphology and exhibit continuous proliferation, resulting in high density than undifferentiated WJ-MSCs. Resulting WJ-MSC-derived SCLSs express typical SC markers, i.e., GFAP, p75, S100β, and MBP. Further, these differentiated cells well support the axonal outgrowth in vitro from DRG neurons (Figure 1 and Table 1) [155].

##### Application in the PNS

Human WJ-MSC-derived SCLCs are seeded on to hollow fibers (Amicon, Beverly, MA, USA) and transplanted into an 8 mm PNI rat model that is co-treated with tacrolimus. Interestingly, SCLCs maintain their phenotype In vivo and contribute to myelin tissue formation around regenerative axons. Furthermore, the motor function of the animals treated with WJ-MSC-derived SCLCs is found to be significantly higher than undifferentiated WJ-MSCs and comparable to human SCs [156].

##### Application in the CNS

WJ-MSC-derived SCLCs still remain to be evaluated in animals for treating PNI/SCI. However, the beneficial effects of undifferentiated WJ-MSC transplantation in SCI are demonstrated by several studies [157,158]. Clinical studies using WJ-MSC transplantation show the positive impact on motor function, self-care ability, and muscular tension of patients with thoracolumbar SCI grade A [159]. The regenerative effects of WJ-MSCs are mainly associated with their paracrine signals [160]. Within this context, we hypothesize that WJ-MSC-derived SCLCs with improved neurotrophic potency may hold the improved capacity for treating SCI lesions.

##### Limitations

UCB-MSCs and WJ-MSCs are isolated from the umbilical cord, and therefore, a large section of the patients who failed to bank their umbilical cord depend on the allograft source. International standards and quality management are required for long-term cell banking of these potential MSCs [161]. However, further evaluation of the WJ-MSC-derived SCLCs for treating PNI and SCI is required in complete detail.

### 2.2. Hair Follicle/Skin-Derived Stem Cells

Cellular homeostasis and regeneration of the mammalian epidermis rely on the variety of precursor cells, which can be found in the epidermis and in the hair follicle epithelium [162]. Hair follicles possess abundant stem cells with easy accessibility. The hair bulge is a well-characterized niche for adult stem cells, i.e., epithelial stem cells, melanocyte stem cells, and neural crest stem cells (NCSCs) [163,164,165,166,167]. The mesenchymal compartment of the hair follicle harbors dermal sheath, dermal papilla, and dermal precursors. These unique populations of epidermal and dermal cells within the hair follicles possess high differentiation potential (Figure 1 and Table 1).

#### 2.2.1. Neural Crest Stem Cells

##### In Vitro Characterization

Hair follicle-derived NCSCs (Hf-NCSCs) are of high interest for regenerative medicine, given their multi-lineage capacity and wider availability [168]. Similar to endogenous SCs, Hf-NCSCs originate from the embryonic neural crest. Thus, Hf-NCSCs are of great choice for generating SCLCs in vitro. Hf-NCSCs are readily accessible in the bulge of hair follicles and can be isolated with high purity for further expansion [169]. Briefly, hair follicles are dissected, and the bulge sections are placed in adherent culture. Due to their migratory ability, Hf-NCSCs emigrate from the bulge explants. These migratory Hf-NCSCs can be expanded and cryopreserved [170]. Differentiation of rat and human Hf-NCSCs can be achieved in vitro using media supplemented with GGF-2, which is known to suppress neural differentiation while promoting glial differentiation [96]. Within 4 weeks, human Hf-NCSCs become S100- and GFAP-positive [168]. Alternatively, a faster differentiation can be achieved when human Hf-NCSCs are treated with BME and RA, followed by manipulation of the WNT, sonic hedgehog, and transforming growth factor β (TGF-β) signaling pathways and further exposure to bFGF, PDGF-BB, FSK, and GGF-2. Within 4 days after induction, Hf-NCSCs change to a more slender and elongated morphology, representing the characteristic feature of SCs. Further, Hf-NCSC-derived SCLCs show enhanced expression of SOX10, KROX20, p75, MBP, and S100β and become mature within 2 weeks of differentiation. Hf-NCSC-derived SCLCs interact with axons and co-localize with myelin in vitro (Figure 1 and Table 1) [169].

##### Application in the PNS

Multiple studies have demonstrated enhanced axonal regeneration and functional restoration when NCSCs are transplanted into the niche of PNI [171,172,173]. Hf-NCSCs transplanted into a 2 mm PNI mouse model become GFAP-positive SCLCs and support the axonal regeneration and innervation [174]. Hf-NCSCs also regulate the neuroinflammatory responses, and Stratton et al. in 2017 showed myelin regeneration by Hf-NCSCs. However, studies demonstrating the effect of Hf-NCSC-derived SCLCs on PNI are still missing.

##### Application in the CNS

When transplanted into an SCI mouse model, murine Hf-NCSCs differentiate into GFAP/CNPase-positive SCLCs, leading to myelin regeneration and improved motor and sensory function [175,176,177]. However, In vivo applications of Hf-NCSC-derived SCLCs are still missing. Given the potential of Hf-NCSCs to differentiate into SCLCs in vitro as well as In vivo, we hypothesize that Hf-NCSC-derived SCLCs may possess high stability and enhanced neurotrophic potency, leading to better performance.

##### Limitations

An extended or prolonged differentiation process involving several weeks and complex procedure is certainly a major drawback. Further, the lack of In vivo studies showing the therapeutic ability of Hf-NCSC-derived SCLCs is the main limitation. However, the high regeneration potential of Hf-NCSCs, as evidenced by enhanced axonal regeneration, myelin, and functional recovery following PNI and SCI, may circumvent extended time required for in vitro differentiation.

#### 2.2.2. Skin-Derived Precursory Cells

##### In Vitro Characterization

Similar to Hf-NCSCs, SKPs can be harvested from skin and hair follicles. Resulting SKPs can be differentiated in vitro into SCLCs [178,179,180,181]. Briefly, single-cell suspension of skin tissue will be achieved by enzymatic, i.e., collagenase digestion, followed by mechanical dissociation and filtration. These cells are then cultured with epidermal growth factor (EGF) and FGF-2. Within 3–7 days, the floating spheres of SKPs are formed. Further expansion can be achieved by the dissociation of these spheres into single cells and by further subculture. SKPs do express the neural marker nestin; however, they lack the expression of the NCSC markers—p75NTR and PSA-NCAM. Further, it has been shown that SKPs could reconstitute the dermis and induce hair follicle morphogenesis. Thus, it is clear that SKPs originate from embryonic mesenchymal precursors [181]. Differentiation of human and rodent SKPs is achieved by culturing dissociated SKP spheres on poly-D-lysine and laminin-coated plates in the presence of FSK and HRG. Within 10 days, the cells become bipolar and express the SC markers, such as S100β, MBP, PMP22, GFAP, and P75NTR. Further, DRG co-cultures reveal the myelination capability of SKP-derived SCLCs in vitro (Figure 1 and Table 1) [178,182].

##### Application in the PNS

SKP-derived SCLC transplantation into the distal segment of a crushed sciatic nerve reveals the association of transplanted cells with regenerating axons and the expression of MBP and PMP22 after 2 weeks. Interestingly, naive SKP transplant shows a similar expression of myelin proteins; however, expression levels appear to be lower than the SKP-derived SCLCs. Interestingly, a subpopulation of SKP-derived SCLCs expresses GFAP but not MBP while aligning with axons, suggesting that these cells may belong to non-myelinating SCLCs [178]. The 12 mm decellularized nerve graft seeded with SKP-derived SCLCs promotes significant loco-motor function when implanted into a 10 mm sciatic nerve gap injury. Seventeen weeks post-operatively, SKP-derived SCLCs accelerate functional regeneration that can be comparable to Sham control groups, as evidenced by the tapered beam task in contrast to other groups receiving isografts, nerve-derived SC, or media alone. Further, the amount of axons, action potential amplitudes, and muscle weights are found to be significantly higher for the animals treated with SKP-derived SCLCs [183].

##### Application in the CNS

SKPs, when transplanted into the brains of newborn shiverer mice, differentiate into myelinating cells, presumably a response to axonal-derived factors within the niche of developing brain. The CNS of shiverer mice is characterized by extensive demyelination. Compact myelin formation is observed following SKP transplantation, further confirming the myelination potential of in vivo differentiated SKPs [178,184]. In ex vivo studies, it has been shown that in vitro SKP-derived SCLCs express MBP and S100β within the cerebellar white matter in cerebellar slice cultures [178]. Immediate injection of rodent SKP-derived SCLCs into the lesion site of an SCI crush model promotes repair and functional recovery within 6 weeks. SKP-derived SCLCs implantation results in increased usage of the injury-affected forelimb, enhanced axonal density in the rubrospinal tract rostral to the lesion, and significant EMG thresholds that are comparable to uninjured animals. Further, no significant differences between nerve-derived SCs and SKP-derived SCLCs are found in several measures, such as motor function, electrophysiological properties, graft survival, neuroprotection, myelination, and integration into the host parenchyma [185]. Transplantation of SKP-derived SCLCs into a rat chronic SCI model reflects a more clinically relevant approach than acute injury. Therefore, SKP-derived SCLCs are transplanted into chronic SCI of rats, i.e., 8 weeks after injury. Subsequent analysis reveals the survival of transplanted SCLCs for 5 months, their integration into host tissue, neural protection, axonal regeneration, and myelination. Further, the functional analysis reveals improved locomotion after 8 weeks of SCLCs transplantation [186].

##### Limitations

Human SKPs display varied gene expression profiles that are subjective to the anatomical region of cell isolation [187,188]. Such heterogeneity is a potential risk factor for their clinical transition. Moreover, there are no markers to distinguish purified human SKPs from the rest of the cells originating from hair follicles [189]. However, by sequential passaging of SKP-derived SCLCs, the purity of the cells could be raised over 95% [178]. Subsequently, it is crucial to study the utility of human SKP-derived SCLCs in order to assess their suitability for clinical applications.

### 2.3. Pluripotent Stem Cells

Pluripotent stem cells, i.e., ESCs, as well as induced pluripotent stem cells (iPSCs), are of great interest for the generation of SCLCs. iPSCs are highly similar to ESCs in terms of gene signature, epigenetic status, and differentiation potential [190,191]. Therefore, iPSCs are attractive autologous cells, represent a viable alternative to ESCs, which are accessible from the inner cell mass of pre-implantation blastocysts only [192,193].

#### 2.3.1. In Vitro Characterization

SCLCs generation from pluripotent progenitors generally relies on an intermediate stage called NCSCs. These NCSCs can be either derived from neural rosettes formed on an MS-5 stromal feeder cell layer or from non-adherent neurospheres that are induced by PA6 stromal cells [194,195]. ESC-derived NCSCs can be further differentiated into neural crest derivatives like sensory and sympathetic neurons, smooth muscle cells, and SCs [196,197]. ESC-derived NCSC differentiation towards SCs can be achieved by various combinations of factors, such as HRG, BDNF, GNF, NGF, FSK, bFGF, and cAMP. Further, myelin formation can be induced by ascorbic acid [198]. Human ESC-derived SCLCs wrap and myelinate rat DRG neurons in vitro [199,200,201]. ESC differentiation into SCLCs involving an intermediate step is a time-consuming process, requiring several weeks to months. Direct differentiation, involving no NCSC stage, can be achieved by inducing ESCs or iPSCs into SC precursors (SCP). First, neural rosettes from human pluripotent stem cells can be derived by modulating the Glycogen synthase kinase 3 (GSK-3) and TGF-β pathways using inhibitors in the absence of feeder cells [202,203]. Next, neuregulin1 (NRG1) induces neural rosettes differentiation towards self-renewing SOX10-positive SCP, which can be further differentiated into immature SCLCs using NRG1, RA, PDGF-BB, and FSK. Resulting cells express trophic factors, such as BDNF, GDNF, NGF, and NT-3, and promote axonal regeneration from rat DRG neurons and deposit the myelin around the axons.

#### 2.3.2. In Vivo Application

ESC-derived SCLCs support the anatomical regeneration and enhanced motor function within 8 weeks after matrigel-assisted transplantation into PNI [204]. Further structural analysis reveals the co-localization of MBP with S100-positive ESC-derived SCLSs, indicating their myelination potential. These observations are further strengthened by accelerated sciatic functional recovery assessed by walking track analysis [204].

#### 2.3.3. Limitations

The therapeutic use of ESCs in clinics is associated with ethical, safety, and regulatory considerations. Within this context, autologous-derived iPSC holds better chances for the clinical transition than ESCs. However, iPSC-based applications are limited due to the increased safety risks associated with the genetic reprograms involving genome-integrating viruses and proto-oncogenes, i.e., c-Myc used for the induction of pluripotency can lead to genomic instability and tumorigenesis [190,205]. Moreover, teratoma formation is reported to be another major risk linked to iPSCs’ clinical applications. Low numbers of undifferentiated ESCs or iPSCs can result in teratoma formation after implantation [30,206]. Thus, there is a need to explore more effective yet safe strategies for the differentiation of ESC/iPSC into SCLCs.

### 2.4. Fibroblasts

Recent studies in the field of cellular reprogramming have demonstrated the feasibility of somatic cell direct conversion into target cell type without passing through a pluripotent intermediate state. Thus, the resulting cells with complete pre-differentiation would be suitable for transplantation therapies without being tumorigenic (Figure 1 and Table 1) [207,208,209].

**Table 1:** Differential origin of Schwann cell-like cells (SCLCs) and their biological performance.

Starting Cell	Induction Factors	Method	Phenotypic Markers	Growth Factor Expression	In Vitro Outcome	In Vivo Outcome	Time (Days)	Subacute/Chronic Injury	Injury	In Vivo Cotreatments	Application in PNS/CNS	Ref.
Ad-MSC	BME, RA, FSK, bFGF, PDGF, HRG	direct biochemical induction	morphology	BDNF, NGF, GDNF	increased neurites sprouting of NG108-15 neurons, increased neurites length and increased amount of neurites per neuron	increased myelination	18 days	subacute	rat tibial crush	-	PNS	[134]
Ad-MSC	BME, RA, FSK, bFGF, PDGF-AA, HRG	direct biochemical induction	-	BDNF, GDNF, VEGF-A, Angiopoietin-1	increased neurites length of rat DRG neurons	increased amount and length of axons, increased angiogenesis	18 days	subacute	10-mm rat sciatic nerve gap	14-mm tubular fibrin conduit; Cyclosporine A	PNS	[126]
Ad-MSC	BME, RA, FSK, bFGF, PDGF-AA, HRG	direct biochemical induction	morphology	BDNF, GDNF, NGF	withdrawel of differrentiation media cause reversion of the induced SCLC phenotype	-	18 days	-	-	-	-	[131]
Ad-MSC	BME, RA, FSK, bFGF, PDGF, HRG, PROG, Hydrocortisone, Insulin	direct biochemical induction	morphology, GFAP, S100, PMP-22, P0	BDNF, NGF	-	increased amount of axons, increased myelination, enhanced motor function recovery	13 days	subacute	10-mm rat sciatic nerve gap	collagen sponge, cyclosporine A	PNS	[143]
BM-MSC	BME, RA, FSK, bFGF, PDGF-AA, GGF-2	direct biochemical induction	morphology, GFAP, S100, p75, erbB3	-	increased neurite sprouting, increased neurite length, increase neurite density of rat DRG neuron	-	18 days	-	-	-	-	[111]
BM-MSC	BME, RA, FSK, bFGF, PDGF-AA, HRG	direct biochemical induction	morphology, GFAP, S100, CNPase, p75NTR, P0	HGF, VEGF	increased number and neurite length of Neuro2A cells	enhanced axonal outgrowth in ex vivo Spinal Cord slices	12 days	-	-	-	CNS (ex vivo)	[112]
BM-MSC	neurosphere induction: bFGF, EGF, B27; SC-like cell induction: FSK, PDGF-AA, bFGF, HRG	two step biochemical induction	morphology, S100, p75	BDNF, VEGF, HGF, NGF	incresed neurites sprouting, increased neurite length of Neuro2A cells and rat DRG neurons, myelination	functional myelination	21 days (neurospheres); 14 days (SC-like cells)	Subacute	5-mm rat sciatic nerve gap	16-mm chitosan conduit; Cyclosporine A	PNS	[121]
BM-MSC	BME, RA, FSK, bFGF, PDGF-AA, HRG	direct biochemical induction	morphology, GFAP, S100, p75, P0	-	-	increased amount of axons, enhanced motor function outcome	8–9 days	Subacute	10-mm rat sciatic nerve gap	10-mm trans-permeable tubes (Hollow fibers, Amicon, Beverly, MA); tacrolimus	PNS	[116]
BM-MSC; Ad-MSC	conditioned SC media	SC co-culture	PMP-22, S100	-	-	-	12 days	-	-	-	-	[97]
ESC	rosette induction: Stromal feeder cells, BME, SHH, FGF8, BDNF, TGFβ, cAMP, ascorbic acid; SC-like cell induction: HRG, CNTF, cAMP	two step biochemical induction: ESC to neural rosette to SC-like cells	GFAP, S100, MBP	-	-	-	16 days (rosette); 60 days (SC-like cells)	-	-	-	-	[201]
ESC	neurosphere induction: Stromal feeder cell, BME; SC-like cell induction: FSK, bFGF, HRG, ascorbic acid	two step biochemical induction: ESC to neurospheres to SC-like cells	morphology, GFAP, S100, p75, PMP-22, P0, MBP, Krox20	-	interaction with chicken & rat DRG neurons	-	14–16 days (neurospheres); 56 days (SC-like cells)	-	-	-	-	[200]
ESC/iPSC	NCC induction: stromal feeder cell, B27, FGF2, Rock inhibitor, ascorbic acid; SC-like cell induction: HRG	two step biochemical induction: ESC/iPSC to NCC to SC-like cells	GFAP, S100, p75, erbB3, Sox9, PMP-22, MBP	-	myelination of rat DRG neurons	-	14 days (neurospheres); 40 days (SC-like cells)	-	-	-	-	[199]
ESC/iPSC	rosette induction: CHIR99021, SB431542; SCP induction: NRG1; SC-like cell induction: NRG1, RA, FSK, PDGF-BB	tree step biochemical induction: ESC/iPSC to rosette to SPCs to SC-like cells	morphology, GFAP, S100, PMP-22, PLP	BDNF, GDNF, NGF, CNTF, NT-3, NT-4	myelination of rat DRG neurons	enhanced myelination, enhanced motor function recovery	6 days (rosette); 18 days (SPC); 7 days (SC-like cells)	suacute	6–9 mm mouse sciatic nerve gap	matrigel	PNS	[204]
Fibroblasts	SOX10, Krox20 transduction; FSK, bFGF, PDGF, HRG	genetic modification	morphology, GFAP, p75, NG2	BDNF, GDNF, NGF	increased neurites sprouting of NG108-15 neurons, increased neurites length, increased amount of neurites per neuron, myelination of mice DRG neurons	enhanced myelination, enhanced motor function recovery	3 days	subacute	5 mm mouse sciatic nerve gap	5 mm gelatin hydrogel conduit	PNS	[207]
Fibroblasts	SOX10, Krox20 transduction; HRG, FSK	genetic modification	morphology, GFAP, erbB3, MAG, P0, MBP		interaction with murine DRG neurons, increased neurites length	-	14 days	-	-	-	-	[210]
Hf-NCC	mouse sciatic nerve	In vivo differentiation	GFAP	-	-	enhanced myelination, enhanced electrical signal transduction		Subacute	2-mm rat sciatic nerve gap		PNS	[174]
Hf-NCC	GGF-2	direct biochemical induction	GFAP, S100	-	-	-	28 days	-	-	-		[168]
Hf-NCC	BME, RA, FSK, bFGF, PDGF-BB, GGF-2, CHIR99021 (GSK inhibitor, WNT activator), SB431542 (TGFβ1 receptor inhibitor)	direct biochemical induction	morphology, S100, p75, MBP, SOX10, Krox20	BDNF, FGF2, FGF5, IL6, VEGF	interaction with murine DRG neurons, myelination	-	4–17 days	-	-	-		[169]
SKP	FSK, HRG	direct biochemical induction	S100, p75, PMP-22, MBP	-	-	integration into CNS white matter in ex vivo spinal cord slices; compact myelin formation in vivo	10 days	chronic demyelination	shiverer mice brain characterized by extensive demyelination		CNS	[178]
SKP	FSK, HRG	direct biochemical induction	morphology, S100, p75, P0	-	myleination of rat DRG neurons	alignement with newly formed myelin	10 days	chronic (implantation 6 days post demyelination)	local demyelination by lysolecthin injection in mice sciatic nerves		PNS	[182]
UCB-MSC	NCC induction: Epidermal Growth Factor, bFGF, B27; SC-like cell induction: RA, FSK, bFGF, PDGF-AA, HRG	two step biochemical induction: UCB-MSC to neurospheres to SC-like cells	morphology, GFAP, S100, Nestin	-	increased neurite sprouting of rat DRG neurons	-	>5 days (neurospheres); 4 days (SC-like cells)	-	-	-	-	[147]
UCB-MSC	BME, RA, FSK, bFGF, PDGF-BB, NGF, HRG	direct biochemical induction	morphology, GFAP, S100, p75	-	-	-	8 days	-	-	-	-	[148]
WJ-MSC	BME, RA, FSK, bFGF, PDGF, HRG	direct biochemical induction	morphology, GFAP, S100, p75, MBP	BDNF, NGF, NT-3	increased neurite sprouting, increased neurite lenght of rat DRG neurons	-	12 days	-	-	-	-	[155]
WJ-MSC	BME, RA, FSK, bFGF, PDGF, HRG	direct biochemical induction	morphology, GFAP, S100, p75, P0, O4	-	-	improved amount of axons, myelination, enhanced motor function recovery	6–7 days	Subacute	8-mm rat sciatic nerve gap	8-mm trans-permeable tubes (Hollow fibers, Amicon, Beverly, MA); tacrolimus	PNS	[156]

#### 2.4.1. In Vitro Characterization

Human fibroblasts can be genetically reprogrammed using lentiviral vectors. Postnatal rat and human skin fibroblasts can be trans-differentiated into S100/O4-positive induced SCs (iSCs) by Sox10 and Krox20 reprogramming [203,206]. Prior to transduction, fibroblasts are placed in the media supplemented with FSK and HRG. The depletion of FSK and HRG in iSCs’ media results in significant cell death, indicating their beneficial effects for the post-transduction survival of iSCs. Transcriptional profiling of resulting cells confirms that iSCs are positive for the SC markers, i.e., MBP, ERBB2, MAG, ERG2, SOX10, myelin protein zero (MPZ), GFAP, and MBP, while being negative for the oligodendrocyte-specific transcription factors and markers, i.e., Myelin oligodendrocyte glycoprotein (MOG), Oligodendrocyte transcription factor 1 & 2 (OLIG1, OLIG2), Neuron-glial antigen 2 (NG2), and NKX2-2. Strong activation and subsequent maintenance of distinct SC factors following transgene transduction clearly indicate the effective reprogramming. Resulting iSCs show potential for myelin formation, as demonstrated by DRG neuronal co-culture experiments. In response to bFGF-stimulation, iSCs exhibit three-fold potency for remyelination, which is closely matching with somatic SCs [210].

#### 2.4.2. In Vivo Characterization

Human iSCs accelerate nerve regeneration and promote motor function within 12 weeks after gelatin hydrogel-assisted transplantation of iSCs into a 5 mm sciatic nerve gap injury in mice [207].

#### 2.4.3. Limitations

The major drawback of genetically modified cells by lentiviral vectors is a random integration of the therapeutic genes that can potentially modify the activity of neighboring genes in close proximity and even inactivates genes completely by integrating into them [211]. Further limitations include ethical and regulatory constraints associated with genetically modified cells.

## 3. Conclusions

PNS and, particularly, CNS regeneration is highly challenging. SC transplantation enables overcoming the hurdles and reversal of the inhibitory microenvironment into a permissive niche. Due to the significant problems associated with SC harvest and culture, the need for stem cell therapy has emerged. Within this context, cells with the following traits, i.e., safety, homogeneity, non-immunogenicity (autologous), wider availability, functional stability, and therapeutic efficacy, are of great interest. ESCs, iPSCs, and genetically modified cells will remain controversial and away from the clinical transition due to ethical, technical, and regulatory constraints. However, the methods to generate SCLCs using ESCs/iPSCs are of great importance for studying gliogenesis and peripheral neuropathies. All other cell types, particularly Ad-MSCs, UCB-MSCs, WJ-MSCs, Hf-NCSCs, and SKPs, hold promise for the clinical transition if provided the possibility of direct and fast differentiation with long-term functional stability and safety. In the clinical settings, the time window between donor cell isolation and patient implantation is of great importance. Following neurotmesis, it becomes absolutely crucial to minimize the time gap for immediate therapy in order to protect the patients from chronic denervation, secondary damage, and comorbidities. Indeed, delayed treatment often results in progressive muscle degeneration and function [13,212]. To overcome these problems, a highly efficient one-step differentiation without intermediate cell stage and extensive cell sorting would be highly desirable. In vitro generation of SCLCs allows vigorous characterization of transplantable cells but requires long-differentiation time. Therefore, the transplantation of progenitor cells, followed by In vivo differentiation, may become attractive and effective in terms of time. Within this context, undifferentiated MSCs hold better chances for the clinical transition in comparison to the differentiated cells that are linked with technical and regulatory hurdles. On the other hand, the development of safe and effective differentiation methods may foster the clinical transition of the differentiated SCLCs with improved therapeutic efficacy.

So far, there are no clinical trials registered using the SCLCs. However, the safety of autologous, in vitro expanded SC transplantation was demonstrated by the Miami clinical trial project in 2017 [29]. Furthermore, there are several studies reporting the therapeutic benefits of MSCs for SCI and other neurological diseases. Intravenous infusion of autologous Ad-MSCs exhibits no serious adverse events. Most importantly, no carcinogenic incidents are reported [60]. In another phase 1 clinical trial, the local injection of autologous BM-MSCs in patients with chronic SCI results in various improvements of tactile sensitivity in all patients. Some patients further gain lower limb motor function [213]. However, in phase 3 clinical trials, the injection of autologous BM-MSCs into chronic SCI only shows improved neurological outcomes in 2 out of 16 patients [214]. Thus, these results remain controversial and do not replicate previous pre-clinical studies. Therefore, the extended pre-clinical studies with clinically relevant models are highly required prior to the clinical studies, and the same is true for SCLCs.

Thus, preclinical studies involving chronic nerve injury models are extremely important in order to mirror the actual clinical scenario. For instance, studying the ways of rejuvenating the niche of chronically denervated distal segments. As shown in Table 1, currently, the studies using human SCLC for treating chronic PNI and SCI are largely missing. Furthermore, the homogeneity of the differentiated SCLCs and the efficiency of their reprogramming procedures are crucial for the success of preclinical and clinical studies.
